# Supplementary material for: Receipt of a combined economic and peer support intervention and clinical outcomes among HIV-positive youth in rural Rwanda: A retrospective cohort
Source: PLOS Glob Public Health. 2022 Jun 28;2(6):e0000492. doi: 10.1371/journal.pgph.0000492 (PMC10021781; doi:10.1371/journal.pgph.0000492)
Supplement: Attachment — Submitted filename: CFAR_ASG_Cohort_R&R_21apr2022.docx [file pgph.0000492.s002.docx]

**Reviewer #1: Thank you for presenting important findings that showed no effect of an intervention on important clinical outcomes. We need to see more evaluations of public health interventions that show no effect so that we can adapt and refine our interventions. The paper is concise and well written. The analysis reported in this paper is as rigorous as it can be using the routine data methods employed and this could result in many biases which you rightly discuss in your limitations. I am happy that although not the "perfect" analysis it is as robust as it can be in the circumstances.**

Thank you very much. We appreciate your supportive view on the benefits and challenges of conducting pragmatic evaluations using existing data. **1. My major concern with the paper is the inability to really learn from the negative findings as the assessment of the intervention is not fully described in this paper. From what is reported this is the subject of another paper, which is referenced but as submitted rather than published, and so the reader cannot access that paper to understand the issues. I would request that a more systematic description of the implementation be included within this paper to make it much stronger. For example were the failures in implementation of the programme as designed uniform across all facilities or were there differences that could be further explored? The intervention is also quite complex and I feel that a table showing the intended design of the intervention and the actual implementation would be helpful to readers and may allow for the differences in fidelity to be better quantified. This would make it easier to understand which aspects were delivered as expected and which not- this is unclear in the current text.**

Thank you for this constructive comment. This intervention was complex, and to fully explore the complexity of the program, our team has or is in the process of producing three papers – these papers are being presented separately because they answer meaningfully different research questions using different data sources and study populations. However, collectively these will allow us to learn from our null result.

The paper to which you refer was led by two colleagues who implemented the program, and it discusses the implementation process and the adaptations that occurred during the intervention. It relies heavily on nurse-reported programmatic data and does not include a control group, which makes it substantively different from the current EMR-based analysis. This paper is now published in an open-access journal, and we have updated our manuscript to include the full citation for this published paper, which would be available to all readers:

Nshimyumuremyi JN, Mukesharurema G, Uwamariya J, Mutunge E, Goodman AS, Ndahimana J d’Amour, et al. Implementation and Adaptation of a Combined Economic Empowerment and Peer Support Program Among Youth Living With HIV in Rural Rwanda. J Int Assoc Provid AIDS Care [Internet]. 2022 Jan [cited 2022 Jan 7];21:232595822110640. Available from: <http://journals.sagepub.com/doi/10.1177/23259582211064038>

A third manuscript drafted will use qualitative interviews to delve into youths’ perceptions around benefits and challenges associated with the ASG. This work will also provide more concrete steps to improve this intervention, and is mentioned in the discussion section on page 17, paragraph 2:

Finally, this analysis only considered clinical outcomes and did not assess economic or psychosocial benefits. **To complement this retrospective cohort, we have conducted qualitative interviews with youth beneficiaries to understand youth perspectives on the program, which we hope will provide insights on why the intervention did not produce the desired clinical impact, what non-clinical benefits may result from participation, and allow us to improve future iterations of this program**. Future programs should consider pairing an improved intervention with a more rigorous, prospective evaluation that includes an appropriate control group and assesses relevant non-clinical outcomes.

However, to provide better context for our readers within the present paper, we have expanded the “Methods: Adolescent Support Group” section of our paper to include additional details on our paper, including Table 1, discussing the incentive structure for the Adolescent Support Group and key adaptations. We have also added a paragraph to our discussion that more explicitly links our findings from the previous paper to the findings of the current cohort analysis on page 16, paragraph 2:

“In our previous assessment on the implementation of the ASG program, we noted that a number of adaptations and infidelities to the program occurred over time [25]. Because recruitment into the ASG program was left to the nurses’ discretion, we observed a much wider range of ages and a much wider range of groups sizes than expected. This variability in implementation contributed to key challenges in the current evaluation. For example – because a large proportion of the ASG members fell outside the target age range of 15-25, we retained youth born between 1987 and 2012 in our analytic sample. This approach avoided excluding an excessively large proportion of ASG members, but also meant that a large proportion of youth in both the intervention and control groups fell outside of the 15-25 target age range. Similarly, in some facilities nurses appeared to recruit almost all youth living with HIV to the ASG program, leaving few youth available to be controls from that sites and preventing us from matching each ASG member with an equal number of control youth. In addition to creating methodologic complications, these infidelities to program implementation could have also made the intervention less successful and partially explain our null findings. Finally, in our previous work where we used programmatic data to assess group-level performance on key indicators, nurses reported much higher ­­group achievement than what was observed in the EMR. For example, over half of the ASG groups reported 100% viral suppressions rates in the first year of the program [25]. There are three likely explanations for this. First, nurses may mis-reported group performance because it was difficult and time consuming to calculate and track indicators, particularly those around viral suppression. Second, nurses could have over-reported group performance in an effort to help the youth at their facility gain access more financial resources. These first two explanations point to implementation issues that could have negatively impacted the program by distorting the link between group behavior and money earned, therefore reducing the motivational power of the incentives. Small adjustments to the award scheme may have made the program easier to implement, better understood among youth, and more in line with behavioral economics theory [32]. For example, rather than incentivizing attendance through the stepwise reward system, promising the group a fixed amount of money each quarter and then implementing penalties for missed appointments might allow the youth to better understand the “cost” of missing appointments. Similarly, providing awards for suppressed viral loads as soon as test results became available would have been both easier to implement and resulted in timelier rewards for good behavior. Finally, incomplete record keeping in the EMR could led us to underestimate attendance and viral suppression; however, we would not expect the process to differ by ASG membership and so it cannot be used to explain our null findings.”

**Reviewer #2: The manuscript by Barnhart and colleagues aims at presenting programmatic outcomes, in Rwanda, linked to retention and to virological success in adolescents who benefited from a dedicated support programme (the Adolescent Support Group (ASG) programme), and adolescents who did not benefit from this dedicated support programme. Briefly, the ASG programme was initiated in 2017 in one region and in 2018 in the two other regions considered. “Economically vulnerable” youth, aged 15 to 24 years of age, were addressed by nurses to the ASG. Each ASG consisted of 10 to 15 members and met once a month to receive peer-support. A key aspect of the ASG was the financial motivation; incentives were deposited in a saving account in case of specific target attainment (i.e. quarterly pharmacy attendance, viral suppression). Then, each member of the ASG received a proportion of the money available on the saving account, depending on the performances of the ASG (100% achievement translated into obtaining 100% of the savings, 90% to 100% achievements translated into obtaining 80% of the savings…). The strategy to offer group-incentives to motivate retention in care is very seducing and worth investigating. However, the article is very unclear and confusing.**
 **1. The study is based on cases and matched controls. However, the choice of the cases is unclear in terms of calendar period and also in terms of timing in the HIV infection. One would expect that controls are the adolescents who were enrolled in the ASG from one date to another date. But there is no such statement in the manuscript. One would then expect the controls to be adolescents not enrolled in the ASG during the same calendar period. But again, the enrolment period is never mentioned.**

**The lack of information regarding the enrolment period is even more surprising as the definition of outcomes are time-dependent; Indeed, the first two outcomes of interest were death-free retention at 12 months, and death-free retention with active follow-up at 12 months. It is unclear what “12 months” refers to in this study. Is it 12 months after enrolment in ASG, after enrolment in the present study, after ART initiation… The same way “active follow-up” is defined as follows: “Patients were under active follow-up at the end of the study period if they were observed <90 days from their last appointment scheduled during the study period or, if a final appointment had not been scheduled after their last observed visit during the study period, <210 days from last observed visit occurring during the study period”. This sentence is very hard to understand, and again refers to the “study period” which is never defined. The section from lines 161 to 176 must be reviewed.**

**The authors mention that controls were matched by facility, year of birth and paediatric infection status. The controls should also be matched by calendar period. Not mentioning this suggests that that participants could be compared over calendar periods that are not similar, and worryingly that the considered calendar period for controls can be before 2017 when ASG were implemented. If this is the case, not considering strictly the same calendar period for cases and controls is a real issue as care conditions were not comparable.**

Your intuition that ASG members and non-members were enrolled during the same time period is correct and we are grateful for the opportunity to make this clearer by the addition of the following bolded text at the bottom of page 5:

**Because the ASG intervention was rolled out in different facilities at different times, we identified specific enrollment periods for each facility**. **At each participating ASG facility, the enrollment period ranging from between one year before the first recorded youth enrolled into the ASG program to one year after the last recorded youth enrolled.** **In several facilities, all youth enrolled in the same date such that this enrollment period spanned two years**; however, other groups embraced a more open enrollment strategy. Patients were considered to belong to the most frequently attended ASG-participating location they visited during that enrollment period. For each ASG members, baseline was considered to start on the patient’s closest observed visit date within 90 days of their recorded enrollment date. Non-ASG members were also assigned a “pseudostart” date selected at random from the distribution program start dates among ASG participants at their health facility or pseudostart date. This pseudostart date was used as the baseline date for subsequent 12-month outcomes.

Although we did not explicitly match on calendar time, ASG members and controls within the same facilities were constrained to have similar start dates, as explained in the new text of the methods section on paragraph 3, page 6:

To improve the comparability of our ASG members and control group, we matched control youth to ASG member within strata defined by health facility, year of birth (categorized as 1987-1992; 1993-1997; 1998-2002, and 2003-2012), and whether or not the patient had initially enrolled the HIV program as a pediatric patient, as a mother in the PMTCT program, or through another route. In our primary analysis, we randomly selected up to two control youth per ASG youth from each stratum. **We did not explicitly match on calendar time; however, because a) non-ASG members were assigned a pseudostart date selected at random from the distribution program start dates among ASG participants at their health facility and b) only individuals with an observed visit date within 90 days of this pseudostart date were eligible to be controls, the distribution of start date for ASG members and eligible controls were constrained to be similar.**

Our definition of 12-month outcomes defined with reference to the closest visit data observed within 90 days of enrollment or within 90 days of the pseudostart date for controls. As is clarified on paragraph 2, page 7:

Our primary outcomes of interest were a) death-free retention in care at 12 months, b) death-free retention with active follow-up at 12 months, c) ≥80% adherence to appointment keeping over a 12-month period, and d) viral load suppression at 12 months (<20 copies/ml). **For all twelve-month outcomes, baseline was considered to occur at the closest visit date within 90 days of their enrollment date (for ASG members) or at the pseudostart date (for controls).**

In addition to better describing the methodologic process, we have also added a sentence to the results section explicitly defining the range of dates observed in each group on paragraph 3, page 8:

**The median start dates within this cohort was June 2018 for ASG members (range: May 2017-Sept 2019) and for control youth (range: April 2017-June 2019).**

**3. In Table 1, it appears that 3 adolescents are not yet on ART. The ASG measures rely on pharmacy visits and viral load assessment. I do not understand how those not on ART can fit in the ASG. This is surprising, and I would recommend removing these 3 individuals.**

It would have been possible for youth to be referred to ASG even if they had refused to initiate ART due to internalized stigma, so we do not believe that it is appropriate to exclude these youth. However, it is probably most likely that these youth were on ART, but that information had not been accurately recorded in the EMR. We have relabeled the “Not initiated” category to “No initiation recorded” and added the following text to our limitations paragraph on page 17, paragraph 2:

Second, EMR data is vulnerable to missing and misrecorded data. Although this limitation is most obvious for our outcome of viral load suppression, which was missing for 20% of youth, incorrect or missing information about visit dates would have also biased our estimates of the active follow-up and visits adherence downward. **Similarly, it is likely that the three youth without data on ART regimen likely had initiated treatment but that this information was not properly recorded in the EMR.**

**4. In the section entitled The adolescent Support Group program, it is mentioned that implementation started in 2017 and 2018 depending on the region, and that adolescents can be enrolled in ASG if aged 15 to 25 years (line 79-81). In the Eligibility section (line 126), it is mentioned that patients born between 1987 and 2012 were eligible. The enrolment period is unspecified, but if we assume the enrolment took place in 2018, eligibility criterion makes that the patients could then be as young as 6 years of age and as old as 31 years. This does not suit the target years 15 to 24. Again, this is very confusing and things must be clarified. Also, what are the reasons for adolescents not to be enrolled in the ASG? Did some decline? Were some not invited? If these two situations exist, it could be useful to distinguish these 2 groups of controls as they may not present the same characteristics.**

As mentioned in the adolescent Support Group program section, the implementation of the program differed from the design, including in the age range of targeted youth. We have emphasized this with the addition of the following text on the bottom of page 3 to the top of page 4:

**Originally, the program was designed so that HIV-program nurses at** each participating health facility were asked to refer economically vulnerable youth between the 15-24 years to the ASG program. **At the start of the program, each health facility founded a single ASG. Groups were allowed to be mixed in terms of both sex and age, but were expected to include 10-15 members and meet once per month.** Although clinicians were instructed to prioritize orphans and out-of-school youth, referrals were ultimately based on clinician judgment and strict enrollment criteria were not enforced. **While not all youth were invited to participate, records on who clinicians chose to invite and whether any invited youth declined to participate are not available. Imperfect implementation led over 20% of the youth falling outside the target age range of 15 to 25 years of age and group sizes ranging from 2 to 33. Group composition was also very heterogeneous, with mean age of group members ranging from 11 to 25 and the proportion of male members ranging from 0-67%.**

**5. It would also be helpful to present group characteristics, in terms of pharmacy attendance and virological success, and in terms of payment received. Were there some ASG with extremely good outcomes and some with poor outcomes? Group performance level could be considered as a potential cofactor.**

Thank you for this constructive comment. Our team has published a previous paper focusing specifically on the implementation process that includes group characteristics and group performance. This paper did explore group performance; however, it used programmatic data recorded by nurse implementers rather than EMR data. This paper is cited and is open access for all readers:

Nshimyumuremyi JN, Mukesharurema G, Uwamariya J, Mutunge E, Goodman AS, Ndahimana J d’Amour, et al. Implementation and Adaptation of a Combined Economic Empowerment and Peer Support Program Among Youth Living With HIV in Rural Rwanda. J Int Assoc Provid AIDS Care [Internet]. 2022 Jan [cited 2022 Jan 7];21:232595822110640. Available from: <http://journals.sagepub.com/doi/10.1177/23259582211064038>

We have added the following text on the bottom of page 3 to the top of page 4 to discuss group heterogeneity:

**Originally, the program was designed so that HIV-program nurses at** each participating health facility were asked to refer economically vulnerable youth between the 15-24 years to the ASG program. **At the start of the program, each health facility founded a single ASG. Groups were allowed to be mixed in terms of both sex and age, but were expected to include 10-15 members and meet once per month.** Although clinicians were instructed to prioritize orphans and out-of-school youth, referrals were ultimately based on clinician judgment and strict enrollment criteria were not enforced. **While not all youth were invited to participate, records on who clinicians chose to invite and whether any invited youth declined to participate are not available. Imperfect implementation led over 20% of the youth falling outside the target age range of 15 to 25 years of age and group sizes ranging from 2 to 33. Group composition was also very heterogeneous, with mean age of group members ranging from 11 to 25 and the proportion of male members ranging from 0-67%.**

We have highlighted the differences observed between the EMR data during this current analysis and the programmatic data reported by implementing nurses and discussing possible reasons for and implications of these discrepancies in paragraph 2 of page 16:

“**Finally, in our previous work where we used programmatic data to assess group-level performance on key indicators, nurses reported much higher group achievement than what was observed in the EMR. For example, over half of the ASG groups reported 100% viral suppressions rates in the first year of the program [25]. There are three likely explanations for this. First, nurses may mis-reported group performance because it was difficult and time consuming to calculate and track indicators, particularly those around viral suppression. Second, nurses could have over-reported group performance in an effort to help the youth at their facility gain access more financial resources. These first two explanations point to implementation issues that could have negatively impacted the program by distorting the link between group behavior and money earned, therefore reducing the motivational power of the incentives.** Small adjustments to the award scheme may have made the program easier to implement, better understood among youth, and more in line with behavioral economics theory [26]. For example, rather than incentivizing attendance through the stepwise reward system, promising the group a fixed amount of money each quarter and then implementing penalties for missed appointments might allow the youth to better understand the “cost” of missing appointments. Similarly, providing awards for suppressed viral loads as soon as test results became available would have been both easier to implement and resulted in timelier rewards for good behavior. **Finally, incomplete record keeping in the EMR could led us to underestimate attendance and viral suppression; however, we would not expect the record keeping among ASG members to be worse than among controls, and so this hypothesis alone cannot be used to explain our null findings.”**

Including group performance as a covariate in our models is not possible because control youth do not have group-performance data. Additionally, adding group achievement to our model would make us vulnerable to collider stratification bias because it is a post-treatment variable.

**6. In the method section, the authors mention “multiple imputation” procedures when dealing with viral suppression outcomes. I am not very comfortable with the fact that outcome are imputed, based on available demographic and clinical characteristics; and then the association between these same demographic and clinical factors and the imputed outcome is investigated. In table 1, the authors state that some demographic characteristics were also missing. Did the authors perform multiple imputations on the demographic characteristics also? This is not clear and this would be wiser to impute these data rather than the outcomes.**

As clarified on paragraph 3 of page 7, the only variable that we imputed was viral suppression. Missing data for other covariates was categorized, as shown in Table 2, and these categories were included in our model. Wherever we present analyses using the multiply imputed data (tables 4 and 5), we have also presented a complete case analysis that does not include any imputed data. In each case, the results do not substantively change between the imputed and not imputed models.

Due to substantial missingness in our viral suppression outcome, we conducted both a complete case and a multiply imputed analysis, which used data on demographic and clinical characteristics to generate 25 imputations. **Imputation was only conducted for the viral suppression outcome. Patients with missing data on covariates were retained in the models using a missing indicator method.** In our models, we dichotomized duration in HIV program (<5 years versus ≥5 years) and ART treatment category (second line versus not second line) due to positivity issues.

**8. In the method section, I do not understand the difference-in-difference analysis. I really do not understand what the authors did.**

At the bottom of page 7, we have an additional reference to readers who may be unfamiliar with this analysis and edited the text as follows to make the motivation for this analysis clearer. We would respectfully request editorial advice as to whether additional information on this type of study design would be helpful or if most readers of PLoS Global Health would be familiar with this method.

**To mitigate this bias, we additionally conducted a difference-in-difference analysis, which is common method for analyzing health programs when randomization is not possible [26]. This analysis was used to compare changes in outcomes among ASG members to changes** in outcomes among control youth in the year before and year after the intervention. To be eligible for this analysis, youth had to have enrolled in HIV care more than 12 months before the start of follow up and have an observed visiting within 90 days of that date. Our difference-in-difference models included fixed effects for being an ASG member versus a control youth, for pre- versus post-intervention status, and their interaction as well as random intercepts for youth and health facility. The interaction term was used to assess the extent to which changes observed among the ASG members after enrollment exceeded changes observed among the control youth over a similar period.

**7. Although it seems the case, it would be useful, for a better understanding of the ASG programme, to know if groups were of mixed gender and mixed age as well.**

We have clarified this with the inclusion of the following text at the top of page 4:

“Originally, the program was designed so that HIV-program nurses at each participating health facility were asked to refer economically vulnerable youth between the 15-24 years to the ASG program. **Groups were allowed to be mixed in terms of both sex and age.”**

**8. To make things easier to read, the description of payment to the members of the ASG, based on the group performances, could be presented in a table.**

Thank you – we have summarized the incentive structure in a new Table 1.

**9. In table 1, “duration in HIV program” is not explicit. Please define what this duration really defines.**

We have added the following footnote to table 2 to clarify that duration in HIV program refers to time since the youth’s first recorded visit to receive clinical care for HIV

**10. In Table 1, “last viral load result” would make more sense if a delay was also considered (e.g. measured within 12 months from enrolment).**

Thank you for this suggestion – we have changed the variable to be “No recent viral load result in the last two years.” This change shifted a large proportion of patients into the “No VL test within two years” category. While changing the coding of the viral load variable, we also discovered a small coding error that changed the categorization of BMI for a small number of youth.

Because the new last viral load test category was associated with ASG membership with a p<0.20, it was included in all of the minimally adjusted models throughout the analysis.

These updated models and categorizations minimally affected many of the point estimates, 95% confidence, and p-values for tables 3, 4, and 5. However, these changes did not meaningfully impact the interpretation of our analyses.

**11. The conclusion of the manuscript seems to be that ASG did not prove useful in terms of retention or virological success. However, the manuscript lacks of clear information, definition and data to support that conclusion. First, the selection procedures and enrolment criteria must be clearly stated, especially in terms of calendar period. Then, I would recommend presenting some ASG outcomes by group and not just by individuals.**

We hope that these edits have fully clarified our enrollment criteria throughout the methods section. For group performance outcomes, which were not assessed using EMR records and are not available for control youth, we would refer you to our previous paper, which discusses these items in great detail:

Nshimyumuremyi JN, Mukesharurema G, Uwamariya J, Mutunge E, Goodman AS, Ndahimana J d’Amour, et al. Implementation and Adaptation of a Combined Economic Empowerment and Peer Support Program Among Youth Living With HIV in Rural Rwanda. J Int Assoc Provid AIDS Care [Internet]. 2022 Jan [cited 2022 Jan 7];21:232595822110640. Available from: <http://journals.sagepub.com/doi/10.1177/23259582211064038>
